# Health status of recreational runners over 10-km up to ultra-marathon distance based on data of the NURMI Study Step 2

**DOI:** 10.1038/s41598-022-13844-4

**Published:** 2022-06-18

**Authors:** Katharina Wirnitzer, Patrick Boldt, Gerold Wirnitzer, Claus Leitzmann, Derrick Tanous, Mohamad Motevalli, Thomas Rosemann, Beat Knechtle

**Affiliations:** 1 Department of Research and Development in Teacher Education, University College of Teacher Education Tyrol, Innsbruck, Austria; 2 Department of Sport Science, University of Innsbruck, Innsbruck, Austria; 3 Research Center Medical Humanities, Leopold-Franzens University of Innsbruck, Innsbruck, Austria; 4 Department of Child and Adolescent Psychiatry and Psychotherapy, LVR-Klinik Viersen, Viersen, Germany; 5 adventureV & change2V, Stans, Austria; 6 Institute of Nutrition, University of Gießen, Gießen, Germany; 7 Institute of Primary Care, University of Zurich, Zurich, Switzerland; 8 Medbase St. Gallen Am Vadianplatz, St. Gallen, Switzerland

**Keywords:** Health care, Medical research

## Abstract

Endurance running is well-documented to affect health beneficially. However, data are still conflicting in terms of which race distance is associated with the maximum health effects to be obtained. Therefore, the aim of this study was to compare the health status of endurance runners over different race distances. A total of 245 recreational runners (141 females, 104 males) completed an online survey. Health status was assessed by measuring eight dimensions in two clusters of health-related indicators (e.g., body weight, mental health, chronic diseases and hypersensitivity reactions, medication intake) and health-related behaviors (e.g., smoking habits, supplement intake, food choice, healthcare utilization). Each dimension consisted of analytical parameters derived to a general domain score between 0 and 1. Data analysis was performed by using non-parametric ANOVA and MANOVA. There were 89 half-marathon (HM), 65 marathon/ultra-marathon (M/UM), and 91 10-km runners. 10-km runners were leaner than both the HM and M/UM runners (*p* ≤ 0.05). HM runners had higher health scores for six dimensions (body weight, mental health, chronic diseases and hypersensitivity reactions, medication intake, smoking habits, and health care utilization), which contributed to an average score of 77.1% (score range 62–88%) for their overall state of health. Whereas 10-km and M/UM runners had lesser but similar average scores in the overall state of health (71.7% and 72%, respectively). Race distance had a significant association with the dimension “chronic diseases and hypersensitivity reactions” (*p* ≤ 0.05). Despite the null significant associations between race distance and seven (out of eight) multi-item health dimensions, a tendency towards better health status (assessed by domain scores of health) among HM runners was found compared to other distance runners. However, the optimal state of health across all race distances supported the notion that endurance running contributed to overall health and well-being.

Trial registration number: ISRCTN73074080. Retrospectively registered 12th June 2015.

## Introduction

As the basic form of human movement, running is the most popular leisure-time physical activity^1^. This low-cost and convenient activity can be practiced at any age with little effort and a lower level of expertise and mastery^2^. Over the past decades, the number of recreational and professional runners has increased across various distances, marathons in particular^3^, and various reasons for actively following a running routine have been reported by runners. While health-oriented purposes have been shown to be the most significant motive for running^1,4^, literature indicates that several motives including but not limited to leisure, hobby, weight control, winning, and social reasons encourage runners to engage in running activities/events^3,5^. Motivations for running could potentially influence the intensity, duration, and frequency of training routines as well as lifestyle behaviors in endurance runners, which together might affect short- and long-term health status^4,5^. Despite the fact that distance runners are depicted as the healthiest fraction of the general population, it has been reported that a “faster and further” dosage fails^6^.

Research indicated that among 26 different kinds of sport, endurance running provides the most favorable health implications^9^. Regular participation in recreational running was found to positively affect body weight (BW), body fat, blood pressure, blood glucose levels, insulin sensitivity, blood-lipid profile, and musculoskeletal health^10–12^. Additionally, running could favorably influence mood, well-being, and mental status^13,14^. Other mental feelings, including fear, depression, worries, anxiety, and anger within the context of an adjustment disorder, might be positively affected following regular endurance running^14^. Distance running contributes to the prevention of chronic diseases by lowering the risks, such as cardiovascular disease (e.g., coronary artery disease, stroke)^15,16^ and different types of cancer^17,18^. As a potential link between running and overall mortality, cardiorespiratory fitness is a strong predictor for morbidity and mortality, and further reduces total mortality from cardiovascular disease, cancer, infections, and other causes^15,19^. Substantial health-related advantages following endurance running are correlated with running exposure in a dose–response association, as the larger effects on health are achieved with increased loads of running^8^. Moreover, evidence supports more beneficial health effects of regular endurance running on cardiovascular risk factors, particularly artery carotid diameter thickness^20^ and low-grade inflammation^21^ compared to irregular endurance running. In addition to the well-established fact that endurance running is an effective tool to improve individual health^7^, regular and long-term involvement in running activities could be a powerful tool to affect public health positively and thus tackle global health problems^8,9^.

It has been shown that marathon runners benefit from a greater metabolic fitness (e.g., insulin response, fasting lipids, fasting insulin), aerobic performance (e.g., velocity at VO_2_max, running economy), exercise metabolism (e.g., lactate threshold), and skeletal muscle levels of mitochondrial proteins compared to sedentary subjects with matched cardiovascular fitness, age, gender, and body mass index (BMI)^22^. Marathon running was found to significantly diminish the risk of coronary plaque prevalence as a result of reducing the relevant risk factors (e.g., hypertension and hyperlipidemia)^23^. In addition to a low incidence of cardiovascular disease, marathoners are shown to have an extended longevity compared to the general population^24^. The favorable health consequences of distance running are not limited to marathoners, as distance runners in lower and higher mileages were also shown to have comparable outcomes. Evidence indicates that ultra-marathoners were healthier and less often sick compared to the general population^25^. Half marathon running was found to positively affect immune cell proportions, pro-inflammatory cytokine levels, and recovery behavior on a short-term basis as a midterm anti-inflammatory effect^26^. Research on 10-km running demonstrates a positive relationship between running and cardio-metabolic health, independent of exercise volume and cardiorespiratory fitness^27^. Furthermore, compared to longer-distance runners, 10-km runners also appear to be at a lower risk of injuries; however, weekly mileage and race distance were identified as risk factors for injuries in endurance runners^7,28^.

Despite the aforementioned advantageous influences, there have been reports of some adverse effects of distance running on health status (e.g., musculoskeletal injuries, unintended weight reduction, cardiovascular abnormalities) that potentially increase with age^28–30^. The increased exercise-induced stress during an ultra-marathon run leads to several pathophysiological changes, such as an increase in acute phase proteins, a decrease in testosterone, an increase in liver values, hemolysis, skeletal muscle cell damage, micro-hematuria, and a loss of bone mass^31^. Ultra-marathoners also tend to suffer from more knee pain, stress fractures, allergies, and asthma than the general population^25^. In addition, intensive and long-lasting endurance running was found to lead arterial changes toward constricting the coronary, cerebral, and peripheral arteries^1,32,33^, which not only affects performance but could also be associated with an increased risk for acute cardiac disorders (e.g., cardiac death, clinical arrhythmias, angina, myocardial infarcts)^34,35^. While older individuals are at a higher risk^36^, the incidence of race-related cardiac arrest was found to be significantly higher in males than female marathoners—although the overall risk is low^34^. It was found that a half marathon running could also significantly increase post-exercise levels of biomarkers related to cardiovascular damage and dysfunction^37^, which is associated with an increased risk for race-related cardiac arrest^34^. Moreover, activation of the inflammatory response and the detoxification process was shown by proteomic profile changes after a half marathon race, and additional pathways associated with immune response, lipid transport, and coagulation were involved^38^. Distance running is also associated with a high risk of running-induced injuries, as approximately half of the active runners reported having more than one injury per year, with excess BW, the weekly mileage, and the race distance recognized as relevant risk factors^7,28^. Furthermore, gastrointestinal complaints (due to decreased exercise-induced mesenteric blood flow)^39^, symptomatic hyponatremia^35^, and exercise-induced asthma, as well as hay fever, are reported in distance runners^39,40^.

In spite of the well-recognized effects of endurance running on different health parameters, there is a paucity of research comparing the health status among different groups of endurance runners. The available health-associated reports did not distinguish different race distances and instead have focused on 10-km runners^41^, half marathoners^26,37,38^, marathoners^12,22,24^, or ultra-marathoners^31,32^. Therefore, the aim of the present study was to investigate the health-related indicators and behaviors of recreational endurance runners and compare their health status across different race distances. It was hypothesized that the health status differs between endurance runners over 10-km, half-marathon, and marathon/ultra-marathon race distances.

## Methods

### Study design and ethical approval

The present study is a part of the NURMI (Nutrition and Running High Mileage) Study and has been conducted following a cross-sectional design^42^. The NURMI study was designed by an interdisciplinary team of scientists and aims to assess and compare recreational endurance runners by sex, race distance, diet type, etc. Data collection was conducted via a series of self-reported online surveys in three separate but subsequent steps. The NURMI Study Step 1 will therefore examine epidemiological aspects (e.g., age, sex, and prevalence of diet type at running events), Step 2 focuses on behaviors considering running training and racing, nutrition, health, etc., and Step 3 investigates running performance linked to diet and sports-psychological parameters.

The subsequent method was introduced in detail elsewhere^10,42,43^, to which the interested readers are kindly referred. The study protocol was approved by the ethics board of St. Gallen, Switzerland, on May 6, 2015 (EKSG 14/145). The trial registration number is ISRCTN73074080.

### Experimental approach and inclusion criteria

Endurance runners in the NURMI study were mostly engaged from German-speaking countries, including Germany, Austria, and Switzerland. Runners were contacted and recruited mainly via social media, websites of the organizers of marathon events, online running communities, email-lists and runners' magazines, as well as via magazines for health, nutrition and lifestyle, trade fairs on sports, plant-based nutrition and lifestyle, as well as through personal contacts.

Participants completed an online survey within the NURMI Study Step 2, which was available in German and English at www.nurmi-study.com. Prior to completion of the questionnaire, participants were provided a written description of the procedures and gave their informed consent to take part in the study. In parallel, physical and psychological information—including the assignment to one of three basic areas of sports (as participants are mainly active in running due to either health, leisure, or performance foci)—motivation and aim of running activities, and details regarding other sports activities to balance for running were obtained to differentiate between a health, leisure, or predominantly performance-orientated approach. For successful participation in the study, the following inclusion criteria were determined initially: (1) written informed consent; (2) at least 18 years of age; (3) questionnaire Step 2 completed; (4) having a BMI < 30 kg/m^2^; and (5) successful participation in a running event of at least a half-marathon distance in the past two years. However, to avoid an irreversible loss of valuable data sets, those who met the inclusion criteria 1–4 but stated being 10-km runners were included as additional participants and were assigned to a further race distance group.

To control for a minimal status of health linked to a minimum level of fitness and to further enhance the reliability of data sets, BMI-associated criteria were implemented in the present study. With a BMI ≥ 30 kg/m^2^, however, other health-protective and/or weight loss strategies other than running are necessary to reduce body weight safely, and could thus potentially affect health-related data. Therefore, participants with a BMI ≥ 30 kg/m^2^ (n = 3) were excluded from data analysis.

### Data clearance and classification of participants

**Figure 1:**
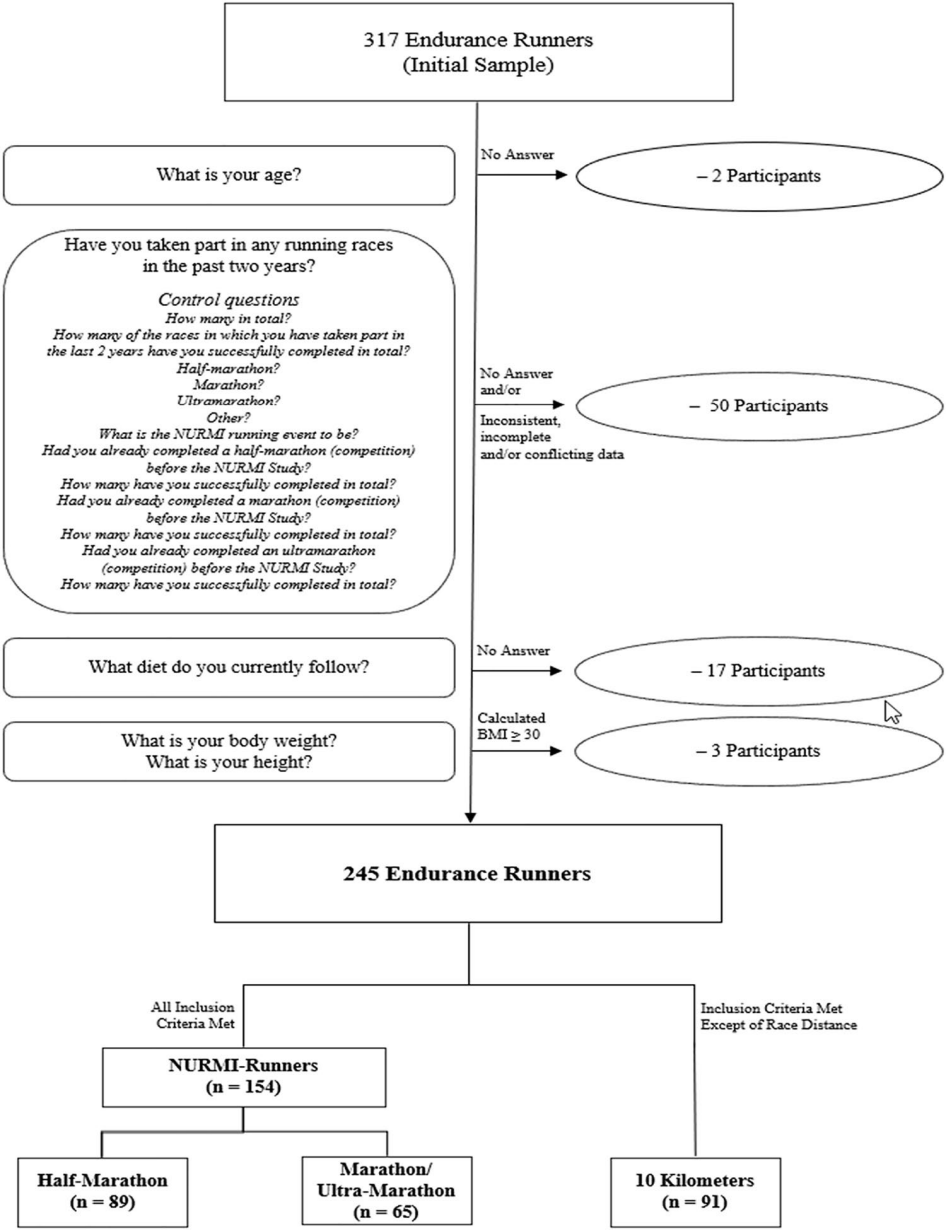
Enrollment and categorization of participants.

Control questions were included throughout different sections of the survey to control for self-reported information of running-related variables (history, training, racing, etc.), and consequently, to identify inconsistent or conflicting data. In general, from the initial number of 317 endurance runners, 72 participants who did not meet the inclusion criteria or did not provide consistent or complete answers to essential questions (e.g., sex, age, race distance, health-related questions) were excluded from the study. As a result, a total of 245 runners with complete data sets were included for descriptive statistical analysis after data clearance (Fig. 1).

Participants were initially categorized according to race distance: half-marathon and marathon/ultra-marathon (data were pooled since the marathon distance is included in an ultra-marathon). The shortest distance for ultra-marathon was 50 km, and the longest distance was 160 km in the present study. In addition, a total of 91 highly-motivated 10-km runners provided accurate and complete answers; however, they had not successfully participated in either a half-marathon or marathon. In general, the most frequently stated race distance was considered the main criterion to assign runners to the respective study groups.

It is well-established that the BMI of active runners is lower than the general population^44^, and people with a higher BMI might have a different health status, as their main goal to engage in running activities is to achieve and maintain a healthy BW. The World Health Organization^45,46^ recommends maintaining a BMI in the range of 18.5–24.9 kg/m^2^ (BMI_NORM_) for individuals, while at the same time pointing to an increased risk of co-morbidities for a BMI 25.0–29.9 kg/m^2^ and moderate to severe risk of co-morbidities for a BMI > 30 kg/m^2^. Therefore, calculated BMI was classified into three categories, under 18.49, BMI_NORM_, and over 25, to differentiate health-related findings based on BMI subgroups. In addition, given the importance of diet types in endurance runners’ health status^10,20^, participants were assigned into three dietary subgroups of omnivores, vegetarians, and vegans^47^.

### Health-related dimensions

As a latent variable, health status was derived by using both the two clusters of health-related indicators and health-related behaviors^10,48^. Each cluster pooled four dimensions defined by specific items based on manifest measures. The following dimensions described health-related indicators: (1) BW and BMI; (2) mental health (stress perception); (3) chronic diseases and hypersensitivity reactions: prevalence of chronic diseases (incl. heart disease, state after heart attack, cancer), prevalence of metabolic diseases (incl. diabetes mellitus 1, diabetes mellitus 2, hyperthyroidism, hypothyroidism), prevalence of hypersensitivity reactions (incl. allergies, intolerances); and (4) medication intake (for thyroid disease, for hypertension, for cholesterol level, for contraception). The following dimensions described health-related behaviors: (1) smoking habits (current and history of smoking); (2) supplement intake (supplements prescribed by a doctor, supplements for performance enhancement, supplements to cope with stress); (3) food choice (motivation, desired ingredients, avoided ingredients); and (4) healthcare utilization and regular check-ups. Together, these eight dimensions described health outcomes. Resulting from this, eight domain scores were derived, which generated scores between 0 and 1. Low scores indicate detrimental health associations, while higher scores indicate beneficial health associations [given as mean scores plus standard deviation and percentage (%)].

### Statistical analysis

The statistical software R version 3.5.0 Core Team 2018 (R Foundation for Statistical Computing, Vienna, Austria) was used to perform all statistical analyses. Exploratory analysis was performed by descriptive statistics (median and interquartile range (IQR)). Significant differences between race distance subgroups and domain scores to describe health status were calculated by using a non-parametric ANOVA. Chi-square test and Kruskal–Wallis test were used to examine the association between race distance subgroups and domain scores with nominal scale variables, and Wilcoxon test and Kruskal–Wallis test (ordinal and metric scale) approximated by using the F distributions. State of health was statistically modeled as a latent variable and was derived by manifest variables (e.g., BW, cancer, smoking). In order to scale the state of health described by the respective dimensions of health, a heuristic index between 0 and 1 was defined (equivalence in all items). In order to test the statistical hypothesis considering significant differences between subgroups of race distance, sex, age, academic qualification, and weekly mileage of running for each dimension, a MANOVA was performed to define health status. The assumptions of the ANOVA were verified by residual analysis. The level of statistical significance was set at *p* < 0.05 (statistical trend: 0.05 ≥ *p* < 0.10).

### Ethics approval

The study protocol was approved by the ethics board of St. Gallen, Switzerland on May 6, 2015 (EKSG 14/145). The study was conducted in accordance with the ethical standards of the institutional review board, medical professional codex and the with the 1964 Helsinki declaration and its later amendments as of 1996 as well as Data Security Laws and good clinical practice guidelines.

### Consent to participate

All participants gave written informed consent prior to the testing procedure.

## Results

### Sociodemographic data

A total of 245 endurance runners (141 women and 104 men) with a mean age of 39 (IQR 17) years and a BMI of 21.72 (IQR 3.50) kg/m^2^ were included for final data analysis. Germany (n = 177), Austria (n = 44), and Switzerland (n = 13) had the majority of endurance runners, but 4.5% of participants (n = 11) were from other countries, including Belgium, Brazil, Canada, Italy, Luxemburg, Netherlands, Poland, Spain, and the UK. There were 154 NURMI-Runners (89 half-marathoners, 65 marathoners/ultra-marathoners) and 91 runners over the 10-km distance. The participants reported following an omnivorous diet (44%), vegetarian diet (18%), or vegan diet (37%). Moreover, with regard to the level of academic qualification, 34% of endurance runners (n = 83) had upper secondary/technical education or a university (or higher) degree. In addition, 67% of endurance runners were married or living with partner (Table 1). The characteristics of the subjects are presented in Tables 1 and 2.

**Table 1:** Anthropometric and sociodemographic characteristics of the endurance runners.

	Total	HM	M/UM	10 km
**Number of Subjects**	100% (245)	36% (89)	27% (65)	37% (91)
**Sex**				
Female	58% (141)	55% (49)	38% (25)	74% (67)
Male	42% (104)	45% (40)	62% (40)	26% (24)
**Age (years) (median)**	39 (IQR 17)	37 (IQR 18)	44 (IQR 17)	37 (IQR 18)
**BW (kg) (median)**	65.0 (IQR 14.2)	65.0 (IQR 13.0)	67.5 (IQR 17.5)	62 (IQR 11.0)
**BMI (kg/m**^**2**^**) (median)**	21.72 (IQR 3.50)	21.97 (IQR 3.28)	22.15 (IQR 3.25)	21.30 (IQR 3.94)
**Diet**				
Omnivorous	44% (109)	44% (39)	51% (33)	41% (37)
Vegetarian	18% (45)	22% (20)	15% (10)	16% (15)
Vegan	37% (91)	34% (30)	34% (22)	43% (39)
**Academic qualification**				
No Qualification	< 1% (1)	1% (1)	–	–
Upper Secondary Education/Technical Qualification/GCSE or Equivalent	34% (83)	37% (33)	40% (26)	26% (24)
A Levels or Equivalent	22% (53)	17% (15)	23% (15)	25% (23)
University Degree/Higher Degree (*i.e.,* doctorate)	34% (83)	30% (27)	28% (18)	42% (38)
No Answer	10% (25)	15% (13)	9% (6)	7% (6)
**Marital status**				
Divorced/Separated	6% (15)	6% (5)	6% (4)	7% (6)
Married/Living with Partner	67% (164)	63% (56)	72% (47)	67% (61)
Single	27% (66)	31% (28)	22% (14)	26% (24)
**Country of residence**				
Austria	18% (44)	17% (15)	20% (13)	18% (16)
Germany	72% (177)	73% (65)	69% (45)	74% (67)
Switzerland	5% (13)	7% (6)	8% (5)	2% (2)
Other	4% (11)	3% (3)	3% (2)	7% (6)

Data are presented as “percentage of prevalence (n)” or “median (IQR)”.

*BMI* body mass index, *BW* body weight, *HM* half-marathon, *IQR* interquartile range, *km* kilometers, *M/UM* marathon/ultra-marathon.

**Table 2:** Characteristics of running activity of the subjects.

	Total	HM	M/UM	10 km
**Number of subjects**	100% (245)	36% (89)	27% (65)	37% (91)
**Basic assignment to areas of sport**				
Sport for Health^a^	10% (23)	8% (7)	5% (3)	14% (13)
Sport for Leisure^b^	54% (133)	64% (57)	37% (24)	57% (52)
Sport for Performance^c^	36% (89)	28% (25)	58% (38)	29% (26)
**Motive for running**				
*Initial Motivation for Running*				
Counteraction to Job	9% (22)	10% (9)	11% (7)	7% (6)
Leisure Activity	4% (11)	7% (6)	5% (3)	2% (2)
Hobby	35% (85)	33% (29)	38% (25)	34% (31)
Weight Maintenance	7% (17)	9% (8)	6% (4)	5% (5)
Weight Loss	18% (45)	17% (15)	15% (10)	22% (20)
Health	19% (46)	19% (17)	18% (12)	19% (17)
Other	8% (19)	6% (5)	6% (4)	11% (10)
*Aim for running events*				
For the Pleasure of Running	39% (90)	40% (35)	47% (27)	32% (28)
Specific Placing	3% (8)	2% (2)	5% (3)	3% (3)
Specific Time	51% (117)	51% (44)	44% (25)	55% (48)
Taking Part is All that Matters	7% (16)	7% (6)	4% (2)	9% (8)
**Completion of running events**				
Total Races Completed Before the NURMI Study (median)	8 (IQR 11)	7 (IQR 11)	10 (IQR 10)	7 (IQR 12)
Races Completed in the Past 2 Years Over Distances (median)	8 (IQR 11)	6 (IQR 11)	10 (IQR 11)	7 (IQR 11)
Half-Marathon	2 (IQR 3)	3 (IQR 4)	2 (IQR 3)	1 (IQR 2)
Marathon/Ultra-Marathon	1 (IQR 2)	0 (IQR 1)	2 (IQR 3)	0 (IQR 1)
**Running Training per week (Mean mileage, km)**				
Low Mileage (≤ 1 km)	17% (41)	26% (23)	5% (3)	16% (5)
Medium Mileage (> 19–36 km)	70% (172)	65% (58)	63% (41)	80% (73)
High Mileage (> 36–100 km)	13% (32)	9% (8)	32% (21)	3% (3)
**Other sports to balance for running**				
*Summer Sports*				
Cycling	53% (130)	55% (49)	57% (36)	49% (45)
Swimming	31% (75)	35% (31)	22% (14)	33% (30)
Hiking/Rambling	31% (75)	33% (29)	32% (20)	29% (26)
Trail/Uphill Running	31% (75)	33% (29)	46% (29)	19% (17)
Triathlon	19% (46)	21% (19)	17% (11)	18% (16)
*Winter Sports*				
Skiing (alpine)	14% (34)	15% (13)	16% (10)	12% (11)
Cross Country Skiing	11% (26)	12% (11)	13% (8)	8% (7)
Snowboarding	7% (16)	9% (8)	5% (3)	5% (5)
Ski Touring	4% (9)	7% (6)	5% (3)	–

Data are presented as “percentage of prevalence (n)” or “median (IQR)”.

*HM* half-marathon, *IQR* interquartile range, *km* kilometers, *M/UM* marathon/ultra-marathon.

^a^Sport for health: Those who take part in sports for health reasons and train 2–3 times a week for 30–60 min at a low to moderate intensity with the aim of maintaining or improving their health.

^b^Sport for leisure: Those who take part for leisure reasons and train 2–5 times a week for 60–90 min at a moderate intensity with the aim of enjoying their free time actively.

^c^Sport for performance: Performance athletes train 3–6 times a week, at moderate to high intensities and assiduously follow a long-term training plan, including assessing their performance, with the aim of ascertaining and improving it and measuring it against that of other athletes in competitions.

The basic assignment of endurance runners to sports areas was 54% for leisure activity, 36% for sports achievement, and 10% for health concerns. The main motivation of endurance runners to start running was for hobby (35%), health (19%), or BW loss (18%). The major goal for participation in running events reported was to achieve a specific runtime (51%) followed by the pleasure of running (39%). As a supplementary physical activity, summer sports (53% cycling, 31% respectively swimming, hiking/rambling and trail/uphill running) were reported to be more prevalent than winter sports.

The median number of events completed in our sample was eight races, and the marathoners/ultra-marathoners finished the highest number of races. Depending on the stage of preparation for the main event and/or season within the course of the year, 70% of runners reported their weekly mileage at a medium volume (19–36 km), while 17% and 13% of runners reported low (< 19 km) and high (> 36 km) volumes, respectively (Table 2).

### Health-related indicators

#### Dimension of BW and BMI

There was a significant difference in BW between race distance subgroups (F_(2, 242)_ = 5.05, *p* = 0.007), with 10-km runners weighing less than half-marathoners and marathoners/ultra-marathoners. However, there was no difference in the health-related item BMI between the subgroups (χ^2^_(4)_ = 1.35, *p* = 0.854) (Table 3). In addition, 10-km runners showed the lowest calculated BMI, while half-marathoners contributed the largest fraction of BMI_NORM_ (85%). Although no significant between-group difference was observed in the dimension of “BW and BMI” (F_(2, 242)_ = 0.84, *p* = 0.433), comparative data showed that half-marathoners had the highest score for the health-related indicator “BW and BMI” (0.69 ± 0.39), and were followed closely by marathon/ultra-marathon runners (0.67 ± 0.39) (Table 4).

**Table 3:** Descriptive and ANOVA results for the eight dimensions of health status displayed by race distance.

Cluster and respective Dimensions	HM	M/UM	10 km	Statistics
**‘Health-Related Indicators’**				
**BW and BMI**				
*BW (kg) (median)*	65.0 (IQR 13.0)	67.5 (IQR 17.5)	62 (IQR 11.0)	F_(2, 242)_ = 5.05, *p* = 0.007
*BMI (median)*	21.97 (IQR 3.28)	22.15 (IQR 3.25)	21.30 (IQR 3.94)	χ^2^_(4)_ = 1.35, *p* = 0.854
≤ 18.49	4% (4)	6% (4)	8% (7)	
18.50–24.99	85% (76)	82% (53)	79% (72)	
≥ 25–29.99	10% (9)	12% (8)	13% (12)	
**Mental health**				χ^2^_(2)_ = 5.83, *p* = 0.054
*Stress Perception*				
Yes	27% (23)	42% (23)	44% (36)	
No	73% (62)	58% (32)	56% (46)	
**Chronic diseases/hypersensitivity reactions**				
*Prevalence of Chronic Diseases*				χ^2^_(4)_ = 4.76, *p* = 0.313
Heart Disease	–	2% (1)	–	
Heart Attack	–	–	–	
Cancer	–	–	1% (1)	
No Diseases	100% (85)	98% (54)	99% (81)	
*Prevalence of Metabolic Diseases*				χ^2^_(10)_ = 13.25, *p* = 0.210
Diabetes Mellitus 1	–	4% (2)	–	
Diabetes Mellitus 2	1% (1)	–	1% (1)	
Hyperthyroidism	–	2% (1)	2% (2)	
Hypothyroidism	7% (6)	7% (4)	4% (3)	
Other Diseases	–	–	2% (2)	
No Diseases	92% (78)	87% (48)	90% (74)	
*Prevalence of Hypersensitivity Reactions*				χ^2^_(4)_ = 8.90, *p* = 0.064
Allergies	22% (19)	25% (14)	35% (29)	
Intolerances	5% (4)	4% (2)	11% (9)	
No Reactions	73% (62)	71% (39)	54% (44)	
**Medication intake (regularly)**				χ^2^_(6)_ = 2.64, *p* = 0.852
Thyroid Disease	7% (6)	11% (6)	7% (6)	
Hypertension	4% (3)	2% (1)	2% (2)	
Cholesterol Level	–	–	–	
Other Medication	2% (2)	4% (2)	6% (5)	
No Medication	87% (74)	84% (46)	84% (69)	
Contraceptives (females only)	12% (10)	5% (3)	20% (16)	χ^2^_(2)_ = 5.93, *p* = 0.051
**‘Health-Related Behaviors’**				
**Smoking habits**				χ^2^_(4)_ = 4.00, *p* = 0.406
Non-Smoker	67% (57)	56% (31)	52% (43)	
Ex-Smoker	32% (27)	42% (23)	45% (37)	
Smoker	1% (1)	2% (1)	2% (2)	
**Supplement intake**				
*Prescribed by doctor*	8% (7)	7% (4)	7% (6)	χ^2^_(2)_ = 0.07, *p* = 0.968
*To boost your performance*				χ^2^_(4)_ = 3.52, *p* = 0.476
Occasionally	16% (14)	11% (6)	9% (7)	
Regularly/every day	2% (2)	4% (2)	1% (1)	
*To cope wit stress*				χ^2^_(4)_ = 6.66, *p* = 0.155
Occasionally	6% (5)	7% (4)	6% (5)	
Regularly/every day	5% (4)	–	–	
**Food Choice**				
*Motivation*				
Because it is healthy	74% (63)	73% (40)	68% (56)	χ^2^_(2)_ = 0.74, *p* = 0.690
Because it is health-promoting	81% (69)	82% (45)	87% (71)	χ2_(2)_ = 1.00, *p* = 0.607
Because it is good for maintaining health	88% (75)	87% (48)	94% (77)	χ2_(2)_ = 2.15, *p* = 0.341
*Avoided ingredients*				
Refined Sugar	66% (56)	58% (32)	70% (57)	χ2_(2)_ = 1.89, *p* = 0.390
Sweetener	82% (73)	64% (35)	82% (67)	χ2_(2)_ = 5.63, *p* = 0.060
Fat in General	38% (32)	44% (24)	51% (42)	χ2_(2)_ = 3.13, *p* = 0.210
Saturated Fats	58% (49)	58% (32)	61% (50)	χ2_(2)_ = 0.21, *p* = 0.899
Cholesterol	42% (36)	45% (25)	48% (39)	χ2_(2)_ = 0.46, *p* = 0.794
White Flour	60% (51)	60% (33)	79% (65)	χ^2^_(2)_ = 8.70, *p* = 0.013
Sweets	62% (53)	51% (28)	72% (59)	χ^2^_(2)_ = 6.29, *p* = 0.043
Nibbles	58% (59)	53% (29)	72% (59)	χ^2^_(2)_ = 6.11, *p* = 0.047
Alcohol	52% (44)	53% (29)	60% (49)	χ^2^_(2)_ = 1.22, *p* = 0.542
Caffeine	38% (32)	25% (14)	39% (32)	χ^2^_(2)_ = 3.04, *p* = 0.219
*Desired ingredients*				
Vitamins	82% (70)	80% (44)	80% (66)	χ^2^_(2)_ = 0.15, *p* = 0.928
Minerals/trace elements	73% (62)	71% (39)	73% (60)	χ^2^_(2)_ = 0.10, *p* = 0.953
Antioxidants	54% (46)	45% (25)	52% (43)	χ^2^_(2)_ = 1.06, *p* = 0.587
Phytochemicals	44% (37)	40% (22)	54% (44)	χ^2^_(2)_ = 2.92, *p* = 0.232
Fiber	71% (60)	69% (38)	70% (57)	χ^2^_(2)_ = 0.04, *p* = 0.980
**Health care utilization**				
Regular check-ups or routine health checks	62% (53)	49% (27)	54% (44)	χ^2^_(2)_ = 2.64, *p* = 0.268

Data are presented as “percentage of prevalence (n)” or “median (IQR)”.

*BMI* body mass index, *BW* body weight, *HM* half-marathon, *IQR* interquartile range, *km* kilometers, *M/UM* marathon/ultra-marathon.

**Table 4:** Domain scores of ‘health-related indicators’ and ‘health-related behaviors’ of endurance runners, displayed by race distance groups.

	Total	HM	M/UM	10 km	Statistics
**Health-Related Indicators**					
*BW and BMI*	0.65 (0.40)	0.69 (0.39)	0.67 (0.41)	0.60 (0.42)	F_(2, 242)_ = 0.84, * p* = 0.433
*Mental Health*	0.63 (0.48)	0.73 (0.45)	0.58 (0.50)	0.56 (0.50)	F_(2, 219)_ = 2.95, * p* = 0.054
*Chronic Diseases/Hypersensitivity Reactions*	0.85 (0.19)	0.88 (0.18)	0.85 (0.19)	0.81 (0.20)	F_(2, 219)_ = 3.31, * p* = 0.038
*Medication Intake*	0.85 (0.36)	0.87 (0.34)	0.84 (0.37)	0.84 (0.37)	F_(2, 219)_ = 0.20, * p* = 0.817
**Health-Related Behaviors**					
*Smoking*	0.79 (0.27)	0.83 (0.25)	0.77 (0.27)	0.75 (0.27)	F_(2, 219)_ = 2.00, * p* = 0.138
*Supplement Intake*	0.90 (0.20)	0.88 (0.23)	0.91 (0.21)	0.92 (0.17)	F_(2, 219)_ = 0.92, * p* = 0.400
*Food Choice*	0.68 (0.22)	0.67 (0.21)	0.65 (0.26)	0.72 (0.20)	F_(2, 219)_ = 1.32, * p* = 0.270
*Healthcare Utilization*	0.56 (0.50)	0.62 (0.49)	0.49 (0.50)	0.54 (0.50)	F_(2, 219)_ = 1.32, * p* = 0.270

Data are presented as Domain Scores and (SD): Low scores indicate detrimental health effects; high scores indicate beneficial health effects (scales: 0–1).

*BMI* body mass index, *BW* body weight, *HM* half-marathon, *km* kilometers, *M/UM* marathon/ultra-marathon.

#### Dimension of mental health

There was no significant association between race distance and mental health (χ^2^_(2)_ = 5.83, *p* = 0.054) (Table 3). However, half-marathoners reported least often to suffer from perceived stress (27%, n = 23). Although no significant between-group difference was observed in the dimension of “mental health” (F_(2, 219)_ = 2.95, *p* = 0.054), comparative data showed that half-marathoners had the highest score with regard to mental health (0.73 ± 0.45) (Table 4).

#### Dimension of chronic diseases and hypersensitivity reactions

There was no significant association between race distance and the prevalence of (1) cardiovascular diseases and cancer (χ^2^_(4)_ = 4.76, *p* = 0.313), (2) metabolic diseases (χ^2^_(10)_ = 13.25, *p* = 0.210), and (3) hypersensitivity reactions (χ^2^_(4)_ = 8.90, *p* = 0.064). However, none of the half-marathoners reported having chronic diseases, and half-marathoners most often reported having no metabolic diseases (92%, n = 78) and no hypersensitivity reactions (73%, n = 62) while having allergies the least often (22%, n = 19), (Table 3). Overall, half-marathoners scored highest significantly with regard to the health-related indicator chronic diseases and hypersensitivity reactions, and it was the only dimension with significant between-group differences (0.88 ± 0.18, F_(2, 219)_ = 3.31, *p* = 0.038) (Table 4).

#### Dimension of medication intake

There was no significant association between medication intake and race distance (χ^2^_(6)_ = 2.64, *p* = 0.852). Furthermore, there was no significant association between race distance and the intake of contraceptives (χ^2^_(2)_ = 5.93, *p* = 0.051) (Table 3). However, half-marathoners most often reported having no regular medication intake (87%, n = 74). Although no significant between-group difference was observed in the dimension of “medication intake” (F_(2, 219)_ = 0.20, *p* = 0.817), comparative data showed that half-marathoners had the highest score with regard to medication intake (0.87 ± 0.34) but were closely followed by two other groups (Table 4).

### Health-related behaviors

#### Dimension of smoking habits

Race distance and current or former smoking were not significantly associated (χ^2^_(4)_ = 4.00, *p* = 0.406) (Table 3). In addition, half-marathoners showed the highest fraction of non-smokers (67%, n = 57). Although no significant between-group difference was observed in the dimension of “smoking habits” (F_(2, 219)_ = 2.00, *p* = 0.138), comparative data showed that half-marathoners showed the best health-related behaviors with regard to smoking habits (0.83 ± 0.25) (Table 4).

#### Dimension of supplement intake

There was no significant association between race distance and (1) supplement intake prescribed by a doctor (χ^2^_(2)_ = 0.07, *p* = 0.968), (2) the consumption of performance-enhancing substances (χ^2^_(4)_ = 3.52, *p* = 0.476), or (3) the intake of substances to cope with stress (χ^2^_(4)_ = 6.66, *p* = 0.155) (Table 3). Although no significant between-group difference was observed in the dimension of “supplement intake” (F_(2, 219)_ = 0.92, *p* = 0.400), comparative data showed that 10-km runners had the highest health scores with regard to supplement intake (0.92 ± 0.17) but were closely followed by two other groups (Table 4).

#### Dimension of food choice

There was no significant association between race distance and motives for food choice (1) because it is healthy (χ^2^_(2)_ = 0.74, *p* = 0.690), health-promoting (χ^2^_(2)_ = 1.00, *p* = 0.607), and good for maintaining health (χ^2^_(2)_ = 2.15, *p* = 0.341); (2) in order to obtain vitamins (χ^2^_(2)_ = 0.15, *p* = 0.928), minerals/trace elements (χ^2^_(2)_ = 0.10, *p* = 0.953), antioxidants (χ^2^_(2)_ = 1.06, *p* = 0.587), phytochemicals (χ^2^_(2)_ = 2.92, *p* = 0.232), and fiber (χ^2^_(2)_ = 2.58, *p* = 0.276); or (3) with regard to the avoidance of the following ingredients (Table 3): refined sugar (χ^2^_(2)_ = 1.89, *p* = 0.390), sweeteners (χ^2^_(2)_ = 5.63, *p* = 0.060), fat in general (χ^2^_(2)_ = 3.13, *p* = 0.210), saturated fats (χ^2^_(2)_ = 0.21, *p* = 0.899), cholesterol (χ^2^_(2)_ = 0.46, *p* = 0.794), alcohol (χ^2^_(2)_ = 1.22, *p* = 0.542), and caffeine (χ^2^_(2)_ = 3.04, *p* = 0.219). However, there was a significant association between race distance and food choice with regard to the avoidance of the following ingredients (Table 3): white flour (χ^2^_(2)_ = 8.70, *p* = 0.013), sweets (χ^2^_(2)_ = 6.29, *p* = 0.043), and nibbles (χ^2^_(2)_ = 6.11, *p* = 0.047), with 10-km runners reporting doing so more often (all three food items) than the other distance runners. Although no significant between-group difference was observed in the dimension of “food choice” (F_(2, 219)_ = 1.32, *p* = 0.270), comparative data showed that 10-km runners had the best health-related behaviors with regard to food choice (0.72 ± 0.20) (Table 4).

#### Dimension of healthcare utilization

There was no significant association between the use of regular health check-ups and race distance (χ^2^_(2)_ = 2.64, *p* = 0.268) (Table 3). Although no significant between-group difference was observed in the dimension of “healthcare utilization” (F_(2, 219)_ = 1.32, *p* = 0.270), comparative data showed that half-marathoners had the highest scores with regard to healthcare utilization (0.62 ± 0.49) while marathoners/ultra-marathoners scored lowest (0.49 ± 0.50) (Table 4).

### Results of the MANOVA

The findings of the MANOVA considering the health status of endurance runners are presented in Table 5, indicating significant differences for the following results: (1) education (academic qualification) had an association with BW and BMI (*p* = 0.004), smoking habits (*p* = 0.005), and supplement intake (*p* = 0.022); (2) race distance had a significant association with the dimension “chronic diseases and hypersensitivity reactions” (*p* = 0.038); (3) there was an association between sex and smoking habits (*p* = 0.048); (4) training (weekly mileage) had an association with food choice (*p* = 0.003); and (5) there was an association between age and healthcare utilization (*p* = 0.002). However, no significant associations were found considering the dimensions of mental health and medication intake.

**Table 5:** MANOVA results for the eight dimensions of health status.

Cluster	Dimension	Subgroup	F	*df*	η^2^	*p*
**Health-Related Indicators**	**BW and BMI**	*Race Distance*	0.39	2	0.00	0.677
		*Sex*	1.17	1	0.01	0.281
		*Age*	0.00	1	0.00	0.999
		*Education (academic qualification)*	5.66	2	0.05	0.004
		*Training (weekly mileage)*	0.23	2	0.00	0.797
	**Mental health**	*Race Distance*	2.97	2	0.03	0.053
		*Sex*	3.43	1	0.02	0.065
		*Age*	1.04	1	0.00	0.310
		*Education (academic qualification)*	0.48	2	0.00	0.619
		*Training (weekly mileage)*	0.95	2	0.01	0.390
	**Chronic diseases/hypersensitivity reactions**	*Race Distance*	3.04	2	0.03	0.050
		*Sex*	0.61	1	0.00	0.435
		*Age*	0.24	1	0.00	0.623
		*Education (academic qualification)*	0.65	2	0.01	0.525
		*Training (weekly mileage)*	0.71	2	0.01	0.492
	**Medication Intake**	*Race Distance*	0.20	2	0.00	0.815
		*Sex*	0.92	1	0.00	0.340
		*Age*	3.05	1	0.01	0.082
		*Education (academic qualification)*	1.43	2	0.01	0.241
		*Training (weekly mileage)*	0.56	2	0.01	0.573
**Health-Related Behaviors**	**Smoking habits**	*Race Distance*	2.08	2	0.02	0.128
		*Sex*	3.96	1	0.02	0.048
		*Age*	1.97	1	0.01	0.161
		*Education (academic qualification)*	5.35	2	0.05	0.005
		*Training (weekly mileage)*	0.25	2	0.00	0.776
	**Supplement intake**	*Race Distance*	1.04	2	0.01	0.356
		*Sex*	1.74	1	0.01	0.189
		*Age*	3.05	1	0.01	0.082
		*Education (academic qualification)*	3.88	2	0.04	0.022
		*Training (weekly mileage)*	0.37	2	0.00	0.686
	**Food choice**	*Race Distance*	1.62	2	0.02	0.200
		*Sex*	0.20	1	0.00	0.655
		*Age*	0.55	1	0.00	0.459
		*Education (academic qualification)*	0.29	2	0.00	0.749
		*Training (weekly mileage)*	6.06	2	0.06	0.003
	**Healthcare utilization**	*Race Distance*	1.37	2	0.01	0.256
		*Sex*	2.86	1	0.01	0.092
		*Age*	9.62	1	0.05	0.002
		*Education (academic qualification)*	1.40	2	0.01	0.249
		*Training (weekly mileage)*	0.11	2	0.00	0.899

*BMI* body mass index, *BW* body weight, *df* degrees of freedom. *F* F-value, *η*^*2*^ partial effect (small: 0.01; medium: 0.059; large: 0.138), *p p* value for between-group differences.

## Discussion

This study aimed to investigate the potential differences in the health status of recreational half-marathoners, marathoners/ultra-marathoners, and 10-km runners. Mental health, BW and BMI, the prevalence of chronic diseases and hypersensitivity reactions, medication and supplement intake, smoking habits, food choice from ingredients to be avoided or desired, and regular or routine health checkups were measured and compared between the study groups. The main findings were (1) that while no association between race distance and seven health dimensions were found, “chronic diseases and hypersensitivity reactions” had a significant association with race distance, and (2) compared to 10-km and marathon/ultra-marathon runners, half-marathoners showed a tendency towards better scores in six out of eight dimensions of health (BW/BMI, mental health, chronic diseases and hypersensitivity reactions, medication intake, smoking habits, and health care utilization) with an average score of 77.1%; the half-marathon distance was found to contribute best to the overall health status among endurance runners.

Interestingly, only 8% of half-marathon runners and 10% of the overall sample reported “sport for health” as the basic assignment to a sports area, while “sport for leisure” (54% of total participants, 64% of half-marathoners) and “sport for performance” (36% of total participants, 28% of half-marathon runners) were ranked higher. “Hobby” and “health” with 34% and 19% of total participants, respectively, were ranked highest among other initial motives for running, with no considerable difference between the study groups. The number of completed races shows that endurance athletes in the present study are not novices but rather active in recreational (not professional) running. It has been shown that recreational participation in running activities could affect some health-related findings^49^, which could be linked to the participants’ slight emphasis on specific personal achievements versus the joy of running (53% vs. 47%) as the main goal to participate in running events. Consistent with the present findings, it has been reported that “the joy of running races” was a top reason, and “winning” was identified as an unimportant reason to participate in running events^4^. Although “health” was the second-highest ranked reason among the seven motivations for running, it could be considered as the 1^st^ rank (by 44%) when pooled with two other health-related motivations (BW loss and maintenance). This finding is consistent with the literature available, with the main underlying intention probably being to achieve the advantageous effects and pronounced benefits associated with health^1,4^, especially for long-term adherence to running activity^4,50^. Running is expected to be a powerful strategy in the prevention of diseases, promotion of health, and maintenance of a good state of health, especially in elderly populations with an age of ≥ 50 years^50^.

### BW and BMI

Four out of five endurance runners in this study were found to have a BW that corresponds to a healthy BMI_NORM_. Half-marathoners most often matched the BMI_NORM_ and consequently had higher health scores compared to marathoners/ultra-marathoners and 10-km runners. However, 10-km runners were found to have lower BW than half- to ultra-marathoners, nicely matching their reports where BW loss was ranked 2nd highest motivation to start running. In addition, the higher score of 10-km runners in food choices compared to runners over longer distances could be partially associated with the existing findings regarding their trend toward having a lower BW. Another justification could be the higher number of vegan runners in 10-km compared to half-marathon and marathon/ultra-marathon groups in the present study.

About 25% of runners in the present study stated BW management (loss: 18%, and maintenance: 7%) as the reason to start running. However, the half-marathoners seem to established a good balance between running-induced energy required and dietary intake, as they reported least often a decrease in BW due to a change in their diet. These findings emphasize the significance of BW control strategies for endurance runners as dietary changes potentially cause unintended BW loss^29,51^, and adherence to appropriate nutrition strategies for sustainable BW management is highly advised to endurance runners^29^. Although the lower BMI and being leaner were found to be associated with increased endurance running performance^52^, and training/competing in longer race distances correlates with a decrease in BW and body fat^53^, evidence excludes marathon runners or ultra-endurance athletes from this fact^54,55^. This is consistent with the present findings where marathon/ultra-marathon runners had a slight but non-significant higher BMI. The higher BMI of ultra-marathon runners compared to shorter distance endurance runners might be due to the lower importance of running speed in long-distance compared to shorter distance runs. In general, however, reports from the successful runners over 10-km and marathon distance indicate that an optimal BMI for health and performance was found to be between 19 and 20 kg/m^2^^56^. The vegan diet was shown to effectively reduce BW and particularly body fat^57,58^, with favorable effects on running performance, if planed appropriately^59^. Consistently, previous data from our laboratory show that vegan endurance runners are significantly leaner than omnivores (64 kg vs. 68 kg), contributing to their overall state of health with the highest health score (69%)^10^.

### Mental health

While most participants were not suffering from mental stress, half-marathoners reported lower perception of pressure and stress compared to 10-km runners and marathoners/ultra-marathoners. In line with the present findings, it has been found that endurance running leads to stress reduction, a better mood, and higher resilience to psychological pressure and anxiety^43,60^. However, data in terms of the appropriate amount of physical activity in order to maximize these positive effects while avoiding negative effects is sparse. Too little exercise does not evoke beneficial effects, but too much exercise (defined as overtraining) can cause the perception of stress^60^. Half-marathon allows performance to increase within a short period of time, which provides the feeling of success^38^. These characteristics are supposed to lead to a certain degree of life satisfaction and thus a resilience to stress and pressure perception^43^.

### Chronic disease and hypersensitivity reactions

The present study revealed a significant difference between the race distance groups and the dimension, “chronic diseases and hypersensitive reactions”, most beneficially contributing to the half-marathoners’ state of health. Recreational endurance running is well accepted, having various health effects with robust evidence for regular running to add benefits in aerobic, metabolic, and cardiovascular function at rest. Consistent with the study findings, running has beneficial influences on the prevention of chronic and cardio-metabolic diseases, including but not limited to coronary heart disease, stroke, hypertension, diabetes mellitus type 2, and hypercholesterolemia, mainly via increasing cardiorespiratory fitness as a strong predictor for morbidity and mortality^8,9,12,15^. This is in line with another finding from the present study, where race distance was found to have a significant association with chronic diseases and hypersensitivity reactions. These exercise-induced advantageous effects are based on various mechanisms, such as adaptations to the cardiorespiratory and cardio-metabolic system (e.g., changes in the musculoskeletal system and heart muscle cells, increased maximal oxygen uptake), modifications in hormonal response and enzymatic activity, the activation of both inflammatory response and detoxification processes, the involvement of pathways associated to immune response, lipid transport and coagulation, and further genetic adaptions^38,61^.

The present findings could be influenced by the distribution of diet types, particularly vegetarians and vegans, among the endurance runners. It has been reported that appropriately planned vegetarian and vegan diets are healthful and nutritionally adequate even for athletes and provide health benefits for the prevention and treatment of cardio-metabolic disorders and certain diseases such as ischemic heart disease, type 2 diabetes, hypertension, inflammatory problems, and some types of cancer^47,62^. More specifically, the higher prevalence of plant diets together with the null association between race distance and the incidence of allergies in the present study is in line with the available data on the protective effects of fruits and vegetables on the incidence of food allergies, including allergic asthma^18^ as well as the lower prevalence of allergies in vegan endurance runners (20%) compared to omnivores (32%) and vegetarians (36%)^10^. Despite the null association between the occurrence of food intolerances and race distance in the present study, gastrointestinal complaints due to food intolerances are common among endurance runners^63^, probably caused by subclinical food sensitivities that occur during vigorous exercise^64^.

### Medication intake

Medication intake in the form of contraceptives was lower with a statistical trend (*p* = 0.051) in marathoners/ultra-marathoners compared to half-marathoners and 10-km runners. This finding, however, could be explained by a sex-based bias as there were fewer females (38%) among marathoners/ultra-marathoners than in half-marathoners (55%) and 10-km runners (74%). Indeed, 85% of those who reported an intake of thyroid hormones were women, and 100% of those who reported an intake of other hormones than thyroid medication were women who reported the intake of contraceptives. However, there was no association between sex and the dimension “medication intake” when runners were pooled for the MANOVA. As a well-established fact associated with the present findings, women suffer more often from hypothyroidism than men^65^, and importantly, more than 100 million women worldwide use contraceptive pills to avoid undesired pregnancies^66^. Although there were no associations between race distance and the intake of any medication, race distance had a considerable association (score range 0.82–0.86) with medication intake. However, as the majority of distance runners (84–87%) reported no medication intake, caution must be considered when interpreting the present limited data concerning the intake of non-contraceptives medications across different subgroups of distance runners.

### Smoking habits

A low rate of smoking (< 2%) was found in endurance runners across all race distances. Consistently, data indicate that smoking prevalence is usually quite low among endurance runners^67^. This can be justified by undesired performance limitations due to smoking^68^ and the health-consciousness of athletes in general^69^. On the other hand, adhering to regular physical exercise, particularly endurance running, can be an effective way to prevent people from smoking or even help in smoking cessation by reducing cessation-related mood symptoms, cigarette cravings, and withdrawal symptoms among temporarily abstinent smokers^68^. In the present study, there was no association between smoking habits and race distance, but half-marathoners showed a better score in this dimension. While no comparable data are available in the literature, evidence has found a positive association between smoking quitters and running activity in terms of weekly training mileage^67^.

### Supplement intake and performance-enhancing substances

The most commonly reported supplement by the runners was vitamin D. Several studies have detected a huge difference between required and real vitamin D intake in athletes worldwide^70,71^. In addition to dietary intake, athletes’ vitamin D level depends on skin color, training day-time, indoor/outdoor training, and geographic location^71^. Although supplement intake was not associated with race distance, it was found to have high scores (score range 0.88–0.92) among race distance groups, with a slight predominance in 10-km runners. However, the prevalence of intake was generally low, reflected by high health scores across all race distance subgroups. Compared with the highest rate of supplement intake reported by half-marathoners (16%), a recent study reported that 30% of female and 40.2% of male endurance runners consume supplements in order to enhance performance^72^. Although few studies have yet compared different groups of endurance runners regarding the patterns of supplement intake^73^, it has been well-documented that endurance athletes use supplements to a greater extent than non-endurance athletes^74^, probably due to the higher exercise-induced nutritional requirements associated with long-time training, competition, and recovery^75^. Reports from a recent study on elite track and field athletes indicated that distance runners have a significantly higher prevalence in supplemental micronutrient but not macronutrient intake when compared to runners in other track and field disciplines^76^. Moreover, there is some evidence for an increasing problem of doping among elite endurance runners^77^. However, as the participants in the present study were mostly recreational runners, they may have different choices of dietary supplements, which could be associated with their different goals for engaging in training and competition compared to elite athletes^49^. In addition, findings from the present study regarding the participants’ attitudes towards food choices characterize them as being health-conscious, so they might have been aware of potential detrimental effects of risky performance-enhancing substances. In general, despite the fact that the beneficial effects of many supplements on the promotion of health, prevention of chronic disease, and enhancement of athletic performance remain unclear^78^, it is well-established that these products significantly contribute to the nutrient requirements of athletes^78–80^.

### Food choice

The present study showed that food choice was not associated with race distance, but the runners over the 10-km distance reported choosing food in order to avoid white flour, sweets, and nibbles more often than half to ultra-marathoners. This is even reflected by their higher score for food choice (72% vs. 67% and 65%) along with their motivation for choosing food based on health-promoting and health-maintaining reasons. However, caution must be warranted while interpreting the findings, as the higher score of 10-km runners in food choice could be potentially associated with their lower BMI among the study groups. Although the majority of the runners in this study reported following a mixed diet, 59% of 10-km and 56% of half-marathon runners reported following vegetarian/vegan diets, which were recently found to add most advantageous benefits to the runners’ state of health mainly due to maximizing favorable food choice behaviors in endurance runners^10^. The imbalanced distribution of vegans in the 10-km group (compared to the overall groups) might explain, in part, the highest scores for both supplement intake and food choice, as vegans are known to be more health-conscious and thus take special care and compensate for potential deficiencies considering critical nutrients such as vitamin B_12_^10,59,81^. Considering a health-related food choice to get desired ingredients by a specific choice of healthy and health-maintaining items, most participants reported health-conscious behavior across all race distance subgroups. This finding was in line with available literature^2,69^, where athletes were characterized as being health-conscious, particularly with regard to food choice^10^.

### Healthcare utilization

Overall, most athletes reported seeing a doctor at least once a year and making use of regular health checkups. These findings were consistent with the previous literature^82^ and emphasize the fact that regular and sustainable physical activity can diminish morbidity rates and thus the necessity for doctor consultations^83^. The endurance runners of the present study were found to have a good balance between healthy physical activity and vigorous exercise, which could be advantageous for gaining the desired health effects^2^, and importantly for the avoidance of the detrimental consequences of overtraining following excessive running or training activities. In the present study, there was a statistically significant association between race distance and age. Interestingly, and although being older than runners over other distances, marathoners/ultra-marathoners had a low score for regular and routine health checkups, indicating disadvantageous contribution to overall health from weak healthcare utilization.

### Limitations, strengths, and future perspectives

There are limitations worth mentioning. The present study shares with others the limitations of the cross-sectional design. The fact that the findings relied on self-reported records should be considered as the primary limitation since under- and over-reporting are potentially prevalent in self-reported data. However, this effect was compensated by using control questions. Also, the high intrinsic motivation of the participants could be consequential to increase the accuracy of their answers to provide a high quality of the data set. The operationalization of state of health as a latent variable (domain scores) should also be considered as a statistical limitation. Nonetheless, the health score was identified as a meaningful tool to assess the health status. In this regard, however, retrospective rating of the cross-sectional design might raise misunderstandings about the associations between health-related variables and race distance, and thus, caution must be warranted in the representativeness of the present findings. Moreover, the sex-based imbalance in the study groups (particularly the higher number of males in the marathon/ultra-marathon group and females in the 10-km group) could be influential on the health-related findings, as females are well-known to be more health-conscious than males considering favorable habits and healthy lifestyles (e.g., physical activity, alcohol/nicotine, plant-based diets). Nevertheless, the data contribute to the growing scientific interest and knowledge in health-related consequences of endurance exercise for distance running in particular, and can be taken as a step towards broadening the body of evidence in the field.

Although it is well-established that endurance running offers various health benefits, the body of science is still contradictory considering both quantity and quality of running activity that enables obtaining the maximum beneficial health effects and preventing the minimum undesired or adverse effects. Therefore, specific knowledge about the interconnectedness of running distance (in training and racing) and health can provide a better basis for athletes, coaches, physicians, and specialists to optimize health-related training and racing strategies. Thus, the results might be useful for different populations by providing such knowledge to aid the decision of an active and healthy lifestyle, with regular involvement in running training, and also to advise individuals to run for sustainable health outcomes. Even at community and public health levels, health authorities can use this information to support policies towards investing in running programs that promote sustainable running training strategies.

## Conclusions

Regardless of the race distance, endurance runners in the present study showed an optimal state of health. This finding supports the notion that endurance running contributes beneficially to an increased level of health. Half-marathon running was found to contribute to 62–88% of their overall state of health; in addition, the higher score of half-marathon runners in overall state of health (77.1% vs. 72.0% in marathon/ultra-marathon runners and 71.7% in 10-km runners), along with the predominance of half-marathoners in six out of eight dimensions, might suggest that recreational runners over the half-marathon distance have a tendency toward a better health status compared to runners over shorter and longer distances. However, among eight health-related dimensions investigated in the present study, only the “chronic diseases and hypersensitivity reactions” dimension was found to have a significant association with race distance, with a significantly better status for half-marathon runners compared to marathoners/ultra-marathoners and 10-km runners.

## Data Availability

The datasets generated during and/or analysed during the current study are not publicly available but may be made available upon reasonable request.
